# Psychosis Crisis Associated with Thyrotoxicosis due to Graves' Disease

**DOI:** 10.1155/2017/6803682

**Published:** 2017-06-11

**Authors:** Lilibet Urias-Uribe, Emmanuel Valdez-Solis, Claudia González-Milán, Claudia Ramírez-Rentería, Aldo Ferreira-Hermosillo

**Affiliations:** ^1^Endocrinology Service, Hospital de Especialidades, Centro Médico Nacional Siglo XXI, Instituto Mexicano del Seguro Social, Ciudad de México, Mexico; ^2^Experimental Endocrinology Investigation Unit, Hospital de Especialidades, Centro Médico Nacional Siglo XXI, Instituto Mexicano del Seguro Social, Ciudad de México, Mexico

## Abstract

We present the case of a patient with previous psychiatric illness, acutely exacerbated by thyroid storm due to Graves' disease, in whom treatment with antipsychotics induced catatonia. These associations are extremely rare and may be confused with Hashimoto's encephalopathy, especially in the presence of anti-thyroid antibodies in cerebrospinal fluid. The treatment consists in the control of the triggering disease (in this case the resolution of the thyrotoxicosis) and the use of benzodiazepines. However, in some cases, the resolution of psychiatric symptoms is partial and may require the use of electroconvulsive therapy.

## 1. Introduction

Thyroid disease is commonly associated with neuropsychiatric manifestations, independently of its etiology. Patients with hyperthyroidism usually complain of irritability, anxiety, lack of concentration, loss of memory, and difficulty in planning daily activities [1]. Less frequently patients develop seizures, myoclonus, chorea, or catatonia and only 1% had a concomitant psychosis's crisis [2]. At this point, Brandt et al. observed that patients with hyperthyroidism had an increased risk of hospitalization due to a psychiatric disease, as well as being in treatment with antipsychotics and anxiolytics [3]. Hu et al. also found that bipolar disorder is common in these patients [4].

Brain et al. first described a 63-year-old male with hypothyroidism that developed disorientation, seizures, and hemiparesis and tested positive for thyroid peroxidase (TPO) autoantibodies in cerebrospinal fluid (CSF) [5]. These neurological manifestations were named Hashimoto's encephalopathy (HE) and it is present in patients with subclinical hypothyroidism, euthyroidism, and overt hypothyroidism but could also appear in patients with hyperthyroidism [6]. Clinical manifestations of HE consist of focal neurological deficits (in 25% of cases) or a diffuse and chronic course characterized by dementia, confusion, and hallucinations (in 75% of cases) [1]. It has been reported that patient's symptomatology improves with steroid treatment and since then it is also known as “glucocorticoid-responsiveness encephalopathy” [7, 8]. Other abnormalities reported are hyperintense lesions in brain magnetic resonance (MR), high protein levels in CSF, and slow EEG-waves [9]. This entity represents a diagnosis challenge and could be easily confounded with a primary psychiatric disease.

We present the case of a woman with previous psychiatric manifestations that get worse due to thyrotoxicosis. Because of her clinical course and the presence of TPO autoantibodies, HE was suspected but finally discarded due to lack of response to steroid treatment. During hospitalization, the patient developed catatonia associated with treatment with haloperidol that finally improved after treatment with methimazole and clonazepam.

## 2. Case Presentation

A 34-year-old woman was taken to emergency room by her relatives, due to “strange behavior,” “judgment alterations,” and suicidal thoughts. They reported a first episode of hallucinations, damage delirium, heteroaggressiveness, and aberrant behavior, developed 12 years before, 6 months after her first delivery. In that occasion, patient was treated with homeopathic drugs (not specified) with apparent remission of symptoms. No further medical evaluation was performed. Five years later, primary hyperthyroidism was diagnosed due to weight loss, body tremor, sweating, and palpitations and treated with methimazole for only three months (patient decided to suspend her treatment). Finally, 3 years before hospitalization, the patient developed a second episode of aggressiveness and isolation. Over again, patient did not go to medical evaluation and her family does not know how much time this behavior remains. At physical examination, she had a heart frequency of 120 beats per minute, heat irradiation, wet skin, lower abdomen pain, and an evident increased volume of thyroid gland. We calculated 45 points at Burch-Wartofsky scale for thyroid storm and laboratory test reported a thyroid-stimulating hormone (TSH) of <0.005 *μ*U/mL (reference 0.27–4.2 *μ*U/mL), free thyroxin level (fT4L) of 7.7 ng/mL (reference 0.9–1.7 ng/mL), a thyroid scintigraphy with increased and diffuse caption, and a general urine test with bacteriuria, with final diagnosis of toxic diffuse goiter with thyrotoxicosis crisis precipitated by urinary tract infection. At this time, we prescribed treatment with quinolones (ciprofloxacin), thionamides (methimazole), beta-blockers (propranolol), cholestyramine, and intravenous steroid (hydrocortisone). Due to neuropsychiatric manifestations, a lumbar puncture was performed obtaining positive thyroid anti-peroxidase autoantibodies. Other autoantibodies were reported as negative (Table 1). We suspected HE; however, despite intensification of treatment with thionamides and steroids, patient persisted with lack of difference to her environment and fluctuation in mental status and with visual and auditory hallucinations. This allows us to discharge that diagnosis. Psychiatry Department then prescribed treatment with haloperidol 5 mg intramuscularly twice a day without improvement of symptoms. Haloperidol was suspended and treatment was started with risperidone 2 mg/day and quetiapine 300 mg/day, also with poor response. Neurology Department discarded infections, autoimmune diseases, toxic manifestations of drugs, and structural alterations (normal brain MR and EEG). However, patient began with mutism, palsy, negativism, increase in general muscle tone, and catalepsy with a positive test for catatonia syndrome according to Bush-Francis Catatonia Screening Instrument (BFSCI, 6 of 22 points) [10]. With this new diagnosis and to rule out neuroleptic malignant syndrome, antipsychotic drugs were suspended and we prescribed lorazepam (3 mg per day) with a notable improvement of symptoms within the first 24 hours [11]. Soon after, patient decreased fT4 level and 15 mCi of radioactive iodine (I^131^) was applied, achieving lower fT4 levels within the first week. Twenty-one days after application of I^131^, the patient started talking and feeding by herself and she was discharged with treatment with quetiapine 300 mg/day and methimazole 10 mg/day. During evaluation in external consult six months after psychosis episode, she remains physically stable (without clinical manifestations of hyperthyroidism) but persists with occasional aberrant behavior. As seen in Table 2, patient persisted with low TSH levels but fT4 were normalized and we finally suspend methimazole.

## 3. Discussion

Psychosis crisis associated with hyperthyroidism is really unusual. In contrast, psychiatric manifestations are commonly associated with hypothyroidism in the form of “myxedema madness” or are related to a quick correction of high levels of thyroid hormones (fT4) [12]. However, even when psychosis could have an independent cause (e.g., a primary disease), thyrotoxicosis by itself could worsen psychiatric manifestations [13]. In fact, it seems that adrenergic hyperactivity observed in patients with hyperthyroidism could influence certain brain functions. Additionally, the decreased levels of transthyretin induce increase on free thyroid hormones in the intracranial space [14].

Differential diagnosis of psychosis crisis must include trauma, autoimmune diseases, drug abuse, iatrogenic causes, strokes, tumors, congenital disorders (velocardiofacial syndrome), metabolic disturbances, sepsis, neurological infections, Addison disease, hyperparathyroidism, temporal lobe epilepsy, NMDA autoantibodies-associated encephalopathy, and schizophrenia [15, 16]. This implies an adequate evaluation by a psychiatrist and a neurologist with specific treatment for each disease.

Psychiatric symptoms in this case were first considered as an atypical psychosis, since the patient exhibits a strong component of disorientation/confusion, catatonic symptoms, and multiple-modalities hallucinations. Initially, we suspected HE due to progressive neurological and psychiatric manifestations with positivity of autoantibodies [17]. At this point, up to 86% of patients with HE have elevated serum anti-TPO titers, 48% have anti-thyroglobulin (anti-Tg) autoantibodies, and 65% have anti-enolase autoantibodies, without correlation between the antibody titer and the severity of the disease [1]. However, clinical data improve after administration of steroids or immunosuppressant, which did not occur in this case. Additionally, the use of antipsychotic drugs in this group of patients may induce neurotoxicity that only responds after administration of benzodiazepines [3].

In this case, a disease evolution greater than six months, previous history of psychiatric illness, and persistence of symptoms despite the improvement of thyroid disease support the coexistence of a primary psychiatric disorder, which was exacerbated by thyrotoxicosis. After reassessment of clinical case, we suspected that psychotic symptoms developed after delivery were caused by postpartum psychosis with a mixed episode in the context of bipolar mood disorder, with subsequent episodes associated with thyroid disease. Women with bipolar disorder are more likely to experience postpartum psychosis (up to 22%) [18]. Additionally, it is not uncommon that patients with bipolar disorders develop catatonic symptoms, specially associated with treatment with some neuroleptics as those prescribed in this case [19]. However, final psychiatric diagnosis in this case is a challenge, due to patient's relative's lack of information of her usual mood (in order to identify a depressive behavior). We speculated that first episode could be induced by transient thyrotoxicosis associated with thyroiditis that also affects some women in postpartum period (caused by postpartum rebound of thyroid antibodies during first month postpartum) [18]. Furthermore, 5% to 7% of thyroid diseases are observed during postpartum period and almost 20% of women with postpartum psychosis had a thyroid disease [20].

Thyroid hormones play an important role in the regulation of mood and cognition, and the spectrum of neuropsychiatric manifestations associated with thyroid diseases is highly broad [21]. As described in this case, the triggering medical cause must be treated without omitting the coexistence of a primary cause. We must consider that psychiatric symptoms are a combination of genetic vulnerability and environmental stress (triggering diseases). Treatment involves an adequate follow-up by Psychiatry and Neurology. At this point, since there is no definitive psychiatric improvement despite resolution of thyroid disease, it is proposed that patient may require electroconvulsive therapy [22].

## Figures and Tables

**Table 1:** Autoimmune assessment in case reported.

Parameter	Patient value	Reference value
Anti-TPO (CSF)	11.96	0–5.61 UI/ML
Anti-dsDNA/ANA	Negative	Negative
Anti-SM and anti-Ro/SSA	Negative	Negative
cANCA	1.12 U/mL	0.00–5.00
Anti-NMDA	Negative	Negative
RF	3.70	<15 UI/mL
IgE	333	0.00–100 mg/dL
IgA	1607	70–400 mg/dL
IgM	340	40–240 mg/dL
CPK	45	26–170 U/L

Anti-TPO: thyroid peroxidase antibodies; CSF: cerebrospinal fluid; anti-DNA: anti-DNA double strand; ANA: antinuclear autoantibodies; anti-SM: anti-Smith; anti-RO/SSA: anti-Sjögren-syndrome-related antigen A; cANCA: cytoplasmic antineutrophil antibodies; anti-NMDA: anti-N-methyl D-aspartate receptor antibodies; RF: rheumatoid factor; Ig: immunoglobulin; CPK: creatine phosphokinase. Except for anti-TPO, all autoantibodies were performed in patient's serum.

**Table 2:** Thyroid profile evolution of case reported.

Time of assessment	TSH (0.27–4.2 *µ*U/mL)	fT4 (0.93–1.7 ng/dL)
Upon admission	<0.005	>7.77
At 15 days		4.03
At 30 days		3.68
At discharge		2.25
2 months after discharge		1.88
4 months after discharge	0.008	0.96

TSH: thyroid-stimulating hormone; fT4: free thyroxin level.
